# Books and Authors

**Published:** 1908-08

**Authors:** 


### BOOKS AND AUTHORS.

**The Blues** (Splanchnic Neurasthenia). Causes and Cure. By **Albert Abrams,** A.M., M.D., (Heibelberg), F.R.M.S., Consulting Physician, Denver National Hospital for Consumptives. 12 mo, pp. 287. Illustrated. Third Edition. New York: E. B. Treat & Co. 1908. (Cloth, $1.50.)

This book in two previous editions has received favorable mention in these columns. Under a new name, it deals with a form of neurasthenia, more or less common in these days of hurry and worry. The additions in this issue are a note on the vasomotor test in splanchnic neurasthenia in the appendix, and a chapter on intestinal autointoxication.

This is the right sort of book to read at odd times,—traveling, resting, or seeking repose between engagements. It is instructive and at the same time not overtaxing to the mental functions or powers.

---

**A Textbook of Surgical Anatomy.** By **William Francis Campbell,** M.D., Professor of Anatomy in the Long Island College Hospital. Octavo, pp. 675. Illustrated. Philadelphia and London: W. B. Saunders Co. 1908. (Cloth, $5.00.)

Whoever would become a good surgeon must acquaint himself with anatomy in a most thorough manner. After acquiring a knowledge of general anatomy, the next important step is to study and specialise, by the application of this knowledge to clinical conditions to be met in the practice of surgery. This book is intended to aid in utilising the facts already established and to make them available in the operating room or at the bedside. Campbell has been known to the professional world for a long time as a capable teacher of anatomy, and it is fitting that he should undertake the preparation of such a work as this.

After a careful examination of the book, we are convinced of its great usefulness and consider it one of the best surgical anatomy's yet published. Especially would we invite attention to the illustrations as being unusually well adapted for instruction in this line. No surgeon, no one who has any just claim to the title of surgeon, will fail to add this book to his library.

---

**Nervous and Mental Diseases.** For Students and Practitioners. By **Charles S. Potts,** M.D., Professor of Neurology in the Medico-Chirurgical College of Philadelphia. Second edition. 12mo, 570 pages, with 133 engravings and 9 full-page plates. Lea & Febiger, Philadelphia and New York. (Cloth, $2.50 net.)

To the student of medicine one of the difficult problems is to acquire the art of diagnosis in nervous diseases. This manual is an excellent aid to the solution of some of the most complex of these questions. Indeed, unless the undergraduate expects to become an expert in diseases of the nervous system, this book will serve his purpose until he equips his library with one or more of the larger or more comprehensive treatises. The arrangement of subjects, the large faced type with which the paragraphs begin, the make-up, paper and binding all appeal to the eye of the expert as a splendid exhibition of excellent shop work. The scientific quality of the text is of the first order and embraces the important advances that have taken place since the first edition was put forth.

---

**Bradycardia and Tachycardia.** Part II. Disorders of Respiration and Circulation. By **Prof. Edmund Von Neusser,** Professor of the Second Medical Clinic, Vienna; Associate Editor of Nothnagel's Practice of Medicine. Translation by **Andrew MacFarlane,** M.D., Professor of Medical Jurisprudence and Physical Diagnosis, Albany Medical College. 12 mo, 150 pages. New York: E. B. Treat & Co. 1908. (Cloth, $1.25.)

Studies relating to the heart's action, and especially regarding the frequency of its beats, have been made with greater accuracy of late, due to increased laboratory opportunities in part and stimulated by the important bearing bradycardia and tachycardia have upon the clinical manifestations of disease. In this monograph are found the essentials of these two variations from the normal heart beat, as occurring in the more important diseases of respiration and circulation. An appendix contains a number of important references relating to the cause of the heart beat, to the Adams-Stokes symptom complex, and to the Adams-Stokes disease.

---

**Mellin's Food Method of Percentage Feeding.** 12 mo, pp. 183. Illustrated. Boston: Mellin's Food Co. 1908.

This is now the season when the summer diarrheas of infants are prevalent and when the utmost care in feeding must be exercised. Not only this, but the physician must possess rare skill and exercise the most perfect judgment in the management of children afflicted with these disorders. The Mellins food method provides the ways and means for percentage feeding without the necessity of making calculations. The tables and formulas presented in this book are the result of more than four years of strenuous work in the laboratories of the Mellin's Food Company, involving some 3,400 fat analyses in which 1,700 quarts of milk were used. This enormous labor was performed under the personal direction of Edward E. Babb, Ph. G., a member of the American Chemical Society, and may be accepted as reliable in the most perfect sense of the word. The medical profession is greatly indebted to the Mellin's Food Company for working out these tables and formulas, and placing them in this convenient book form at the disposal of physicians. The Mellin's Food Company will appreciate correspondence from physicians relating to the method of percentage feeding as detailed in this book, as well as on the general subject of infant feeding with Mellin's food.

**Progressive Medicine,** Vol. X, June, 1908. A Quarterly Digest of Advances, Discoveries and Improvements in the Medical and Surgical Sciences. Edited by **Hobart Amory Hare,** M.D., Professor of Therapeutics and Materia Medica in the Jefferson Medical College of Philadelphia. Octavo, 352 pages. Lea Brothers & Co: Philadelphia and New York. (Per annum in four cloth-bound volumes, $9.00; in paper binding, $6.00; carriage paid to any address.)

This number opens with a splendid article on hernia by Coley, which is well illustrated. The next topic is surgery of the abdomen in general exclusive of hernia, prepared by Foote. The third article is on gynecology by Clark; the fourth embraces diseases of the blood, diathetic and metabolic diseases; diseases of the spleen, thyroid gland, and lymphatic system by Stengel; and the fifth relates to ophthalmology and is prepared by Jackson. The volume is of excellent quality through and through and will add much to the reputation of this standard periodical.

---

**A Manual of the Diseases of Infants and Children.** By **John Ruhrah,** M.D., Clinical Professor of Diseases of Children in the College of Physicians and Surgeons, Baltimore. Second edition. 12mo, pp. 423. Philadelphia and London: W. B. Saunders Co. 1908. (Limp leather, $2.00.)

This excellent manual has received the commendation of the JOURNAL in its previous editions. Some minor changes have been made, chiefly in the interest of accuracy. The new sections relate to the medical inspection of school children, the duration of the danger of contagion after infectious diseases, and on the return to school of children after exposure to contagious diseases, Also a short bibliography of the current pediatric literature has been added. In its present form it is to be adjudged as one of the better manuals for the medical student. Its value as an aid to the study of the diseases of children cannot be overestimated.

